# Helping Her Heal-Ghana: A pilot feasibility study of a culturally adapted educational counseling intervention for spouse caregivers of women with breast cancer

**DOI:** 10.1017/S1478951524002153

**Published:** 2025-04-07

**Authors:** Brenda Adei Osei-Assibey, Frances Marcus Lewis

**Affiliations:** 1 School of Nursing, University of Washington, Seattle, WA, USA; 2 Public Health Sciences Division, Seattle, WA, USA; 3 Clinical Sciences Division, Fred Hutchinson Cancer Center, Seattle, WA, USA

**Keywords:** Breast cancer, spouse intervention, spousal adjustment, skills training, cognitive-behavioral counseling

## Abstract

**Introduction:**

Breast cancer is the leading cancer in Ghana, Africa, accounting for 31% of all cancers in women. The effects of breast cancer are not limited to the woman but also impact the spouse’s anxiety, depressed mood, and coping behavior. Helping Her Heal (HHH)-Ghana is a culturally adapted evidenced-based intervention with potential to improve health outcomes of spouse caregivers.

**Objectives:**

The purpose of the study was to ascertain the feasibility, acceptability, and short-term impact of HHH-Ghana, a culturally adapted evidenced-based intervention for spouses of women with breast cancer in Ghana.

**Methods:**

The study used a single group pre–post design. Participants (*n* = 14) were recruited from medical care providers and were eligible if they were spouse caregivers of wives with Stage I, II, or III breast cancer, were 18 years or older, and had been living with their wives for at least 6 months. Data were obtained by spouse self-report on standardized measures of depressed mood, anxiety, self-care skills, self-efficacy to support their wife, self-efficacy to carry out their own self‐care, and the quality of marital communication about breast cancer. Exit interviews were additionally obtained to describe the gains spouses attributed to their participation in the study.

**Results:**

The HHH-Ghana study was feasible and acceptable. Spouses actively engaged in each intervention session and completed the at-home assignments; retention was 87.5%. Spouses significantly improved on standardized measures of anxiety (*p* = 0.010), depressed mood (*p* = 0.002), self-care skills (*p* = 0.006), and their self-efficacy in supporting their wife (*p* = 0.001) and in carrying out their own self-care (*p* = 0.011). Although there was no statistically significant change in marital communication, spouses reported in their exit interviews that the intervention enabled them to communicate better and be more attentive listeners to their wives.

**Significance of results:**

Results warrant a larger clinical trial in Ghana.

## Introduction

Globally breast cancer is the leading cancer in women with an estimated incidence of 2.3 million cases (Sung et al. 2021). In Ghana, breast cancer is the leading cancer accounting for 31.4% of all cancers in women. The number of new cases in Ghana in 2021 is 5026 (Sung et al. 2021).

Suffering during breast cancer is not limited to the woman but extends to the spouse and is often referred to as a couple’s disease (Charvoz et al. 2016). During breast cancer, women in Ghana and elsewhere turn to their spouses for support in carrying out activities of daily living and for emotional and financial support (Berger et al. 2019; Boamah Mensah et al. 2020; Kusi et al. 2020), all of which cause strain, burden and weariness in spouses (Gabriel et al. 2019; Neris and dos 2014; Overcash et al. 2019; Rha et al. 2015). Spouses also suffer from anxiety, depression and fatigue due to caregiving responsibilities (Bamgboje-Ayodele et al. 2020; Congard et al. 2019; Janda et al. 2017).

Some spouses in the United Kingdom are known to neglect themselves, their self-care, and their own wellbeing in the process of caregiving and become exhausted (MacLeod 2011). Family caregivers including spouse caregivers are less likely to engage in any form of self-care (Rha et al. 2015) and have reported unmet needs such as making time for self-care (Badr 2017).

A breast cancer diagnosis affects communication in the marital relationship, which places additional demands on the relationship (Keesing et al. 2016). Spouses have difficulty in talking about the breast cancer (Neris and dos 2014), and there is a documented association between observed spouses’ avoidance in communication and anxiety, depression and stress when their wife had breast cancer (Yu and Sherman 2015). Spouses are also more likely to be depressed if they are in less well-adjusted marriages (Lewis et al. 2008a). Spouse confidence in their ability to talk about cancer strengthens the marriage, reduces couple’s stress, and improves mental health (Chen et al. 2021; Magsamen-Conrad et al. 2015). Spouses’ self-efficacy in talking about cancer with their wives predicts the couple’s ability to cope with cancer (Magsamen-Conrad et al. 2015), and evidence show that spouses who have lower self-efficacy in caregiving have more depressive symptoms (Yeung et al. 2020).

Despite all the challenges a breast cancer diagnosis brings to a couple, there are only 2 known interventions directly delivered to spouse caregivers of women with breast cancer (Duggleby et al. 2017; Lewis et al. 2019), even though there are a growing number of interventions that have been conjointly delivered to the couple (Baucom et al. 2009; Budin et al. 2008; Çömez and Karayurt 2020; Fergus et al. 2022; Heinrichs et al. 2012; Kayser et al. 2010; Nicolaisen et al. 2014). Some of these interventions have shown efficacy in reducing anxiety and depression (Lewis et al. 2019; Nicolaisen et al. 2018) and improving the quality of life of couples (Kayser et al. 2010). See Table 1 for a summary of these studies. None of the interventions in Table 1 were tested in low resource environments like Africa or Ghana.

**Table 1.** Characteristics of studies and study outcomes of couple- and spouse-focused interventions

Study and location	Study design	Sample and characteristics	Measures used (Cronbach αs)	Outcome of intervention
Fergus et al., 2022 Canada	Randomized controlled trial with wait list control group	67 dyads <50-year-old patients with non‐metastatic, invasive breast cancer or ductal carcinoma in situ within the last 36 months and their partners.	Positive dyadic coping (PDC; 0.90); Revised Dyadic Adjustment Scale (0.86); Kansas Marital Satisfaction Survey (0.93); Breast Cancer and Relationship Measure (0.86); Hospital Anxiety and Depression Scale (HADS; 0.83 and 0.79 for anxiety and depression subscale).	Modest improvement seen in positive dyadic coping but not sustained at 3 month follow up and no observed between group effects for relationship adjustment. PDC and HADS-anxiety were sensitive.
Lewis et al., 2019 US	Randomized controlled trial	322 dyads Stage 0–III BC	Center for Epidemiologic Studies‐Depression (0.892 for caregivers and 0.894 for patients); State‐Trait Anxiety Inventory (0.935 for caregivers and 0.945 for patients); Mutuality and Interpersonal Sensitivity Scale (spouses’ open communication subscale for the study sample were 0.92 and 0.86, respectively, and 0.88 and 0.82 for the expressing sad feelings subscale for wives and spouses, respectively);What I Do for Her Checklist (0.64 for wife support subscale) and (0.51 for self‐care subscale); Cancer Self‐Efficacy Scale (total scale was 0.952 for the study sample and 0.949 for the wife‐focused and 0.810 for the self‐care subscales); What He Does for Me Questionnaire (completed by wife, 0.88).	At 3 months spouses in the treatment group had improved on anxiety, depressed mood, cancer related marital communication, interpersonal support, and self-care. All differences except anxiety and depression were sustained at 9 months.
Duggleby et al., 2017 Canada	Randomized controlled trial, mixed methods, concurrent feasibility study	40 dyads Patients had stage I–III BC Partners mean age = 55.4 years.	Herth Hope Index (Test-retest r = 0. 91, p = <0.05, Validity, r = 0.84, p = <0.05, Criterion, r = 0.092, p = <0.05, Divergent, r = −0.73, p = <0.05); General Self-Efficacy Scale (0.91), test–retest reliability r = 0.82); Caregiver Guilt Questionnaire (0.93); Caregiver Quality of Life Index – Cancer (Test–retest reliability r = 0.95, internal consistency r = 0.91); Functional Assessment of Cancer-Breast (0.93); Male Transition Toolkit Evaluation Questionnaire was used to evaluate ease of use of the program	Nonsignificant treatment effects on all measures.
Nicolaisen et al., 2017 Denmark	Randomized controlled trial	198 dyads Patients with newly diagnosed with breast cancer Partners mean age = 57.4 years	Impact of Event Scale (0.89 = 0.92 for patients and 0.83–0.89 for partners); Hospital Anxiety and Depression Scale (0.78–0.87 for patients and 0.79–0.84 for partners); Revised Dyadic Adjustment Scale (0.77–0.93 for patients and 0.83–0.94 for partners).	Cancer related distress, anxiety, and depression reduced within the groups but there were no significant intervention effects. There was a significant sustained improvement on the Revised Dyadic Adjustment Scale.
Heinrichs et al., 2012 Germany	Randomized controlled trial (superiority trial) Comparing 2 interventions	90 dyads Patients recently diagnosed with BC and gynecologic cancer and their partners Partners mean age = 52.7 years.	Questionnaire on Stress in Cancer Patients (0.87); Fear of Progression Questionnaire (0.87); Dealing with Illness Inventory-Revised (0.54 and 0.51 for women and men, respectively); Posttraumatic Growth Inventory (0.92 and 0.91 for women and men, respectively); Quality of Marriage Index (0.95); Partnership Questionnaire (0.86 women and 0.82 men).	Superiority of the intervention is limited to fear of progression, avoidant coping, posttraumatic growth, communication, and dyadic coping.
Kayser et al., 2010 US	Randomized controlled trial	Patients with primary, nonmetastatic breast cancer within the last 3 months Partners mean age = 48.7.	The Functional Assessment of Cancer Therapy–Breast (0.92 for the entire scale and alphas ranging from 0.78–0.86 for the 4 subscales); Quality of Life Questionnaire for Spouses (0.94) and the Illness Intrusiveness Rating Scale (0.91).	Quality of life of women and their partners improved in the intervention group but the difference between the 2 groups was not statistically significant.
Baucom et al., 2009 US	Pilot feasibility study	14 dyads Patient had Stage I or II breast cancer	Quality of Marriage Index (0.97 and 0.93 for women and men, respectively); Derogatis Inventory of Sexual Functioning (0.76 and 0.05 for women and men, respectively); Brief Symptom Inventory-18 (0.90 and 0.91 for women and men, respectively); Posttraumatic Growth Inventory (0.97 and 0.96 for women and men, respectively); Functional Assessment of Cancer Therapy Breast (0.70 for women); Self-Image Scale (0.90); Brief Fatigue Inventory (0.90 for women); Brief Pain Inventory (BPI); Rotterdam Symptom Checklist (0.74 for women).	No significant mean differences between intervention and control on any measures in patients or partners. There was improvement on measures within group. Derogatis Inventory of Sexual Functioning and Quality of Marriage Index recorded the most improvement.
Budin et al., 2008 US	Randomized clinical trial with 4 groups (1 control and 3 intervention)	249 dyads Patient has a breast lesion with a confirmed or strongly suspected diagnosis of cancer. Partners mean age = 51.6	Psychosocial Adjustment to Illness Scale; Profile of Adaptation to Life Clinical Scale; Self-rated Health subscale and Breast Cancer Treatment Response Inventory. Internal reliabilities were described as “excellent” for all measures.	Overall, regardless of group assignment, significant main effects for time were seen for both patients and partners in several outcome variables. Partner scores significantly improved over time in physical symptoms and social adjustment.
Scott et al., 2004 Australia	Randomized controlled trial with 3 treatment conditions	Patient about to begin treatment for a primary (localized) breast or gynecological cancer Partners mean age = 53 years	Dyadic Adjustment Scale; Brief Index of Sexual Functioning. “Good” internal consistency was reported on all subscales. Client satisfaction questionnaire was also used.	There was no between group treatment effect although there was within group improvement in coping in the treatment group.

Helping Her Heal (HHH) was developed to improve spouses’ communication and reduce breast-cancer related tension between the spouse and patient and to improve spouses’ anxiety, depressed mood, and self-care. It was efficacy tested in the US and shown to significantly reduce anxiety and depression and improve marital communication, spouse’s self-efficacy and skills in self-care compared to spouses in the control group (Lewis et al. 2019).

The HHH is a spouse-focused intervention based on Bandura’s Social Cognitive Theory (Bandura et al. 1999) and the relational model of adjustment to breast cancer (Ben-Zur et al. 2001; Fang et al. 2001; Hilton et al. 2000; Lewis 2004; Northouse and Swain 1987). It involves 1:1 delivery (by telephone, ZOOM, or in person) and consists of 5 intervention sessions. The intervention sessions are fully scripted with each session having the same internal structure: short educational presentations delivered by the patient educator to the spouse, skills building and efficacy enhancing exercises, and brief at home assignments to be completed by the spouse with his wife.

In a previously completed study, HHH was adapted to the Ghanaian culture and renamed HHH-Ghana (see Table 2). The aim of the current study is to test the feasibility, acceptability, and short-term impact of the culturally adapted intervention with spouses of women with breast cancer in Ghana.

**Table 2.** Session-specific descriptions of Helping Her Heal-Ghana

**Session 1: Anchoring yourself to be strong for her.** This session invites the spouse to describe his experience with his wife’s breast cancer and how he is dealing with it, including what is working and not working for him. The session assists him learn and practice stress-reducing strategies and associate stress reduction with his improved ability to support his wife.	**Specific objectives** Identify the effects of their wife’s breast cancer on their lives. Identify how their own stress changes their interactions with their wife. Identify ways to unwind. Plan to use at least a strategy to unwind for 10–15 minutes each day.
**Session 2: Listening and not fixing: Being the sweetest superman.** This session helps refine the spouse’s skills to be a highly attentive listener for his wife and her breast-cancer-related concerns. Skilled listening involves 3 distinct components, all of which are taught and practiced with the patient educator.	**Specific objectives** Identify how it feels to have someone listen to them. Identify their own behaviors or statements that would demonstrate to their wives that they are listening. Identify how their role as listener differs from their role as fixer, problem solver, and comforter. Learn through enactment of a 3-part listening strategy.
**Session 3: Gaining a deeper understanding of her.** This session builds on Session 2 but focuses the spouse on more advanced skills in eliciting and helping his wife elaborate her concerns or feelings about breast cancer, particularly when she is reticent or withdrawn. These skills help spouses discover new information about their wife’s breast cancer and how to support her.	**Specific objectives** Identify open-ended and closed-ended questions. Examine the benefit of using open-ended questions. Construct open-ended questions. Create open-ended questions about their wife’s breast cancer.
**Session 4: Connecting with her: Creating special times** This session focuses on 3 new nonverbal strategies the spouse can use to increase and enhance the quality of interpersonal connection between him and his wife despite the breast cancer.	**Specific objectives** Identify additional strategies to emotionally connect with their wife by using any of the 2 below: Appreciating her. Using touch. Taking a vacation from the breast cancer.
**Session 5: Putting the pieces together** The final session adds to the spouse’s skills to identify ways to continue to use the strategies he gained from the program. The spouse reflects on what he did and gained, thereby enhancing his self-confidence to manage in future situations.	**Specific objectives** Identify strategies from the program the spouse wants to use in future. Examine personal gains from participating in the program.

## Methods

The study used a single group, pre–post design to assess the feasibility, acceptability and short-term impact of the culturally adapted HHH-Ghana. Ethical approval was given by the University of Washington Human Subjects Division, the Institutional Review Board of the Korle-Bu Teaching Hospital (KBTH) and the Ethics Committee of the Sweden Ghana Medical Center (SGMC). The KBTH is the national referral center located in Accra, the capital city of Ghana. The SGMC, also in Accra, is a private health facility providing specialized cancer care to patients. Sixteen participants were recruited from the KBTH and the SGMC through their diagnosed wives being treated there, recruitment flyers posted at vantage points in the clinic, referral from nurse intermediaries at the 2 centers, and by the first author when approached by potential participants at the 2 facilities. Spouses were eligible if they were married by law or coinhabiting with their partner for at least 6 months, could read and speak English, and their partner had been diagnosed with stage I, II, or III breast cancer within the recent year. Potentially eligible spouses who gave approval to be approached were given details of the study, and invited to ask questions about the study, including the time required for their participation, after which they gave signed informed consent.

Baseline measures were obtained, after which the first author delivered Session 1 of the intervention. The remaining 4 sessions were held 1, 2, and 3 weeks apart based on spouses’ availability and schedule. The intervention was delivered in a private room in one of the facilities or in the participant’s home. Two participants opted for Zoom meetings while 1 participant had 3 sessions in person and 2 sessions on Zoom. All intervention sessions were audio recorded and evaluated for dosage and fidelity using a performance checklist for each of the 5 intervention sessions. See Table 3 for examples of items used for evaluating Session 2. Audio recordings of all 5 sessions of the first 3 participants and 4 other randomly selected participants were reviewed by the first author against the checklist to assess dosage and fidelity. Post-intervention measures were obtained immediately after completing Session 5, the last session of the intervention.

**Table 3.** Performance checklist for some items in session 2

**Session 2: Listening and not fixing: Being the sweetest superman** *Directions for scoring*: In the space before each item, mark: **2** if behavior is present; **1** if behavior partially present (behavior is ineffective or misleading); **0** if behavior is absent or misinformation is given, and **NA** if not applicable.	
Checking-in	
Item	Score
1. Asks man what success he had in finding time to unwind	
2. Invites man to talk about what got in the way of taking time to unwind.	
3. Asks man how it was to take time for himself.	
4. Encourages man to continue to take at least 10–15 minutes a day to unwind.	
5. Invites man to read through his wife’s/partner’s responses to questions for the homework Assignment #1: Anchoring Yourself to Be Strong for Her.	

### Measures

Depressed mood was measured with the Center for Epidemiologic Studies-Depression (CES-D) scale (Radloff 1977). The scale measures the frequency of symptoms of depressed mood experienced within the past week. It is a 20-item self-report 4-point Likert type scale ranging from rarely (0), some (1), most (2), and almost all the time (3). A score of 16 or higher indicates more symptoms of depression. The internal reliability consistency has been well established to be 0.80–0.90 (Radloff 1977) and 0.85–0.90 in other studies (Given et al. 2004; Milette et al. 2010).

Anxiety was measured with the state anxiety subscale of the State-Trait Anxiety Inventory (STAI) (Spielberger 1983). The state anxiety subscale is a 20-item scale that evaluates feelings of apprehension, nervousness, and worry right now. It is a self-report measure consisting of a 4-point interval scale ranging from not at all (1), somewhat (2), moderately so (3), and very much so (4), with higher scores indicating higher anxiety. A score of 40 or higher indicates anxiety. The internal reliability consistency was well established as 0.90 (Spielberger 1983) and 0.935–0.94 in subsequent studies (Edwards and Clarke 2004; Lewis et al. 2019).

Communication within the couple was measured by the Mutuality & Interpersonal Sensitivity Scale (MIS) (Lewis 1996). The MIS is a 32-item self-report measure that assesses the content and ways by which couples communicate about breast cancer. The measure consists of 2 subscales: (1) open communication, “We spend a lot of time talking about how things are going with the breast cancer” and (2) expressing sad feelings, “Sad thoughts about the breast cancer only make things worse.” Response to the questions ranged from always true (5), occasionally true (4), sometimes true (3), seldom true (2), and never true (1). A higher score indicates a better quality of communication about the cancer within the couple. The internal consistency reliabilities for spouses’ open communication and expressing sad feelings subscale from a previous study were 0.86 and 0.82, respectively (Lewis et al. 2019).

Spouses’ skills in supporting their wives and engaging in self-care were assessed with the What I Do for Her Checklist (Lewis et al. 2019). Self-report items describe the specific communication and interpersonal support skills the spouse carries out related to breast cancer. The wife support subscale contains 6 items and measures spouses’ ways of behaviorally interacting with her about the cancer, “I listen calmly to my wife when she tells me sad or negative things about her breast cancer.” The self‐care subscale has 6 items and measures spouses’ ways of coping with their own cancer‐related stress, “I have specific things I do to keep myself calm when my wife talks about fearful things regarding her breast cancer.” Responses range from never (1), once in a while (2), some of the time (3), most of the time (4) and all of the time (5). Higher scores indicate better communication and interpersonal skills. The internal consistency reliability was 0.64 (wife support subscale) and 0.51 (self‐care subscale) (Lewis et al. 2019).

Spouses’ self‐efficacy was measured by the Cancer Self‐Efficacy Scale (CASE), a 19‐item self‐report questionnaire that measures spouse’s degree of self‐confidence to support his wife and carry out his own self‐care (Lewis 1996; Lewis et al. 2008b). The scale contains a wife-focused subscale and a self-focused subscale. The wife-focused subscale (14 items) measures spouses’ confidence in talking with their wife about her cancer‐related concerns and being supportive to her, “I know how to ask my wife questions that help her talk about the breast cancer.” The self‐care subscale (5 items) measures spouses’ confidence in helping themselves cope with the demands and challenges of the breast cancer, “I know what to do to be emotionally supportive to my wife about the breast cancer.” The measure is scored on a scale of 1–10 with 1 being “not at all confident” and 10 indicating “very confident.” A higher score indicates a higher degree of self-confidence of the spouse to support his wife and carry out his own self‐care. The internal consistency reliability from the clinical trial of the HHH for the total scale was 0.95, 0.95 for the wife‐focused scale, and 0.81 for the self‐care subscale (Lewis et al. 2019).

### Data analysis

Prior to analyzing study data, data were inspected for sampling distributions (mean, mode, median), outliers, and floor and ceiling effects. The small sample required the use of nonparametric statistics. There were no outliers or floor or ceiling effects. The impact of the intervention was tested according to a per protocol analysis. Data were analyzed using the Wilcoxon Signed Rank Test, a nonparametric equivalent of a paired t-test. Statistical significance was set at 0.05, 2-tailed tests.

Feasibility was determined by spouse attrition (percent of enrolled spouses who completed the 5 sessions and provided baseline and 3-month post-baseline measures); ease of enrollment; reasons and timing of attrition; number of spouses recruited; number of spouses enrolled; and reasons for eligible spouses declining participation. The ease with which spouses were enrolled was determined by recording the number of times an eligible spouse was contacted before they enrolled.

Acceptability was determined by the spouses’ reported burden in completing questionnaires; their completion of in-session exercises; completing homework; and their feedback about the program immediately at end of Session 5 through exit interviews.

### Qualitative data analysis

The exit interviews were audio recorded and transcribed verbatim. The accuracy of transcripts was ensured by comparing them to the audio recordings. Inductive content analysis was used to code the interview data using methods adapted from grounded theory and described by Lewis and Deal (1995) and most recently by Zahlis et al. (2020). Category labels using words from spouses were used to organize the inductive content analysis, complemented by quotes that represented categories and subcategories (Hsieh and Shannon 2005). Trustworthiness was ensured in 3 ways (1) Constant comparative analysis was carried out throughout the coding process in which coded interview data were reviewed to ensure that each verbatim unit of data was coded into 1 unique category. (2) Peer debriefing was carried out by the second author. (3) An audit trail of word documents of stages of coding process was maintained (Shenton 2004).

## Results

### Feasibility

A total of 34 potentially eligible spouses were recruited to the study, 24 through referral from the nurse intermediary, 6 through the student investigator, and 4 through wives. Sixteen consented to participate in the study, giving an enrollment rate of 47%. The remaining 18 spouses declined due to tight work schedules, the number of sessions involved, or wives not wanting to be discussed. Fifty percent of spouses enrolled after the first initial contact, which was either a personal meeting or a phone call. An average of 3 attempts were made for the remaining participants to enroll. Referral from the nurse intermediary was the most effective and efficient way to identify participants with 10 participants being enrolled through this means. Once enrolled, the retention rate was 87.5%; 14 out of 16 participants completed all 5 sessions of the study.

### Acceptability

Participants completed the study questionnaires in 30–50 minutes, and all participants completed the measures with minimal assistance. However, the majority (10/14) of participants complained that there were too many measures. Some participants initially expressed concern about their being able to complete all 5 sessions. However, once session 1 was delivered, spouses reported the potential usefulness of the intervention to them and attended all the sessions, actively engaged in the sessions by providing responses to questions and completing at home assignments with their wives.

### Short-term impact

**Figure 1.**
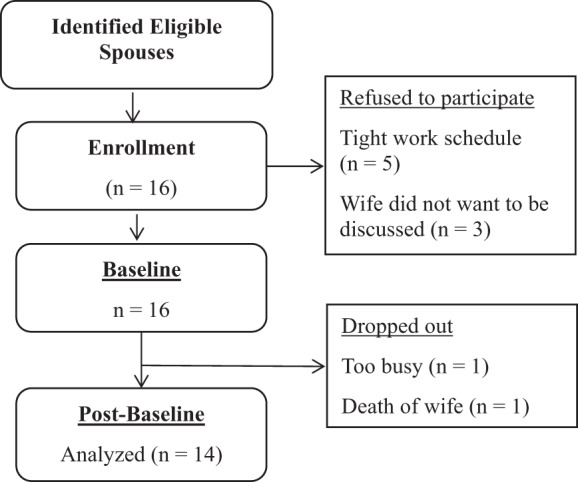
Participant flowchart.

A total of 16 spouses were enrolled (consented and completed baseline data). After enrollment, 2 participants withdrew from the study, 1 was not able to make time for scheduled appointments and the other had his wife die (see Figure 1). There were no differences between drops and completers on demographic and baseline data.

### Description of enrolled study sample

A total of 14 spouses completed the HHH-Ghana Program. See Table 4 for a summary of their sociodemographic characteristics. Some tribes and ethnic groups in Ghana were represented in the study sample, the majority of whom were Akan (*n* = 5) and Ewe (*n* = 5) spouse caregivers.

**Table 4.** Sociodemographic characteristics of participants

Sociodemographic characteristics	Spouse participant	Wife
Age (Mean/SD)	49.21/8.76	44.29/8.17
Employment status		
Employed	13	11
Unemployed	–	3
Retired	1	–
Educational level		
Primary/Basic	1	4
Secondary	2	3
Tertiary/University	11	7
Tribe/Ethnicity		
Akan	5	9
Ewe	5	3
Ga-Dangme	2	1
Dagomba	1	1
Zamrama	1	–
Stage of wife’s breast cancer		
I	N/A	6
II	N/A	4
III	N/A	4
Treatment received by wife		
Chemotherapy	N/A	14
Radiation therapy	N/A	5
Surgery	N/A	10
Time since diagnosis (Mean)	N/A	10 months
Length of marriage (Mean)	17 years	
Number of children (Range)	1–4 children	

### Comparison of pre and posttest scores on spouses’ measures of functioning

There were statistically significant improvements on all but one of the standardized measures of spouse functioning (see Table 5). Measures of depressed mood (CES-D) and anxiety (Spielberger State-Trait Anxiety Inventory (STAI-Y) significantly diminished, *p* = 0.002 and *p* = 0.010, respectively. Self-efficacy (CASE) significantly improved on the self-care (*p* = 0.011) and wife-focused subscales (*p* = 0.001). Spouses’ skills significantly improved on the wife-support subscale (*p* = 0.049) and the self-care subscale (*p* = 0.006).

**Table 5.** Pre and posttest comparisons on outcome measures

	Mean (SD) *n* = 14	Median	*p*-value
*Mood and anxiety*			
CES-D depressed mood			
Pretest	19/11.74	17	0.002
Posttest	7.5/6.39	6	
STAI-Y state anxiety			
Pretest	45.07/14.85	40.5	0.010
Posttest	30.21/10.48	27.5	
***Self-efficacy***			
CASE total scale			
Pretest	141/28.83	149	0.002
Posttest	172.29/12.23	174	
CASE wife-focused subscale			
Pretest	97.71/21.16	103	0.001
Posttest	119.21/7.81	121	
CASE self-care subscale			
Pretest	35.5/9.57	38.5	0.011
Posttest	43.71/4.71	44.5	
***Marital quality***			
MIS-total			
Pretest	116.71/16.84	117.5	0.530
Posttest	118.57/10.52	116	
MIS-open-communication subscale			
Pretest	31.5/7.08	33	0.949
Posttest	32.35/4.40	32	
MIS-Avoid bad thoughts subscale			
Pretest	26.71/7.63	26	0.900
Posttest	26.86/8.65	24.5	
***Spouse behavioural skills***			
Wife support subscale			
Pretest	19.71/3.20	19	0.0498
Posttest	22.29/2.89	22.5	
Self-care subscale			
Pretest	17/3.94	16.5	0.006
Posttest	19.71/4.32	19.5	

*Note*: Wilcoxon Signed Ranks Test; 2-tailed test. CES-D = Center for Epidemiologic Studies-Depression, STAI-Y = State Trait Anxiety Inventory, CASE = Cancer Self-Efficacy Scale, MIS = Mutuality & Interpersonal Sensitivity Scale.

There were no statistically significant changes in the MIS, the cancer-specific measure of marital communication. Neither the total scale nor subscales significantly improved. See Table 6 for a comparison of baseline scores from the current study and those obtained in a previously published pilot study with primarily White spouse caregivers (Lewis et al. 2008a).

**Table 6.** Comparison between baseline scores of HHH-Ghana and HHH-pilot study

	Study	
	HHH-Ghana mean /Median *n* = 14	HHH-pilot mean /median *n* = 20
**Mood and anxiety**		
CES-D depressed mood		
Pretest	19/17	11.40/8.5
STAI-Y state anxiety		
Pretest	45.07/40.5	33.90/34
***Self-efficacy***		
CASE wife-focused subscale		
Pretest	97.71/103	87.05/84
CASE self-care subscale		
Pretest	35.50/38.5	30.74/31
***Marital quality***		
MIS-total		
Pretest	116.71/117.5	105.32/105.5
***Spouse behavioral skills***		
Wife support subscale		
Pretest	19.714/19	22.26/23
Self-care subscale		
Pretest	17/16.5	16.63/16

*Note*: CES-D = Center for Epidemiologic Studies-Depression, STAI-Y = State Trait Anxiety Inventory, CASE = Cancer Self-Efficacy Scale, MIS = Mutuality & Interpersonal Sensitivity Scale.

### Changes in spouses scoring in the clinical range

Comparisons were made between pre and posttest scores on measures with well-established clinical cutoff scores for distress: depressed mood (CES-D ≥ 16) and state anxiety (STAI-Y ≥ 40). We examined whether spouses scoring in the clinical range at baseline (pretest) showed improved or decreased functioning at posttest. We also examined whether spouses scoring within a normal range at pretest backslid at exit from the program.

At baseline, 8 spouses (57%) scored in the clinical range of distress on depressed mood and 8 (57%) on state anxiety. Of the 8 spouses scoring in the clinical range on depressed mood at baseline, only 1 spouse remained in the clinical range at posttest (Fisher’s Exact test *p* = 1.00). None of the spouses scoring in normal range on depressed mood at pretest backslid into the clinical range at posttest. Of the 8 spouses in the clinical range on anxiety, only 1 (the same participant who remained in the clinical range for depressed mood at posttest) remained in the clinical range at posttest (Fisher’s Exact test *p* = 1.00). One of the 6 spouses in the normal range on state anxiety backslid at exit from the program.

### Spouse exit interviews

Inductive analysis of exit interviews revealed 3 categories and 14 subcategories (see Table 7). Each category is more fully described below.

**Table 7.** Categories and subcategories from exit interviews (*n* = 14)

Category	Subcategories
Helping us	Improving my mood Being heard Paying attention to self Being in a better position to support wife Supporting wife Improving relationship with wife Wife not feeling neglected
Improving understanding	Learning new things Adding to what I know Teaching me what to do and not do Understanding wife Understanding breast cancer
Communicating better	Improving communication with wife Listening to wife

### Helping us

Spouses claimed that after their wife’s diagnosis, neither the nurses nor doctors paid attention to them. They were left on their own with their uncertainties and anxieties. Spouses claimed the program helped them and their wives by improving their mood and mental well-being.

I think it’s a therapy because I’ve seen it as going through some kind of an exercise to help me mentally, you know, redress some of the challenges we were dealing with (Participant 16).

Spouses also said the program gave them an opportunity to be heard.

And after that nobody cares about me again. So I think with this program at least you will feel that somebody cares about you as well (Participant 8).

Aside from being heard, spouses felt the program enabled them to gain skills in paying attention to themselves and being in a better position to support their wives. One of the spouses said he had even neglected his own health previously but that has changed due to the program, saying,

I’m always thinking about her alone without checking myself. I have an eye problem, but I was not going for my checkups. But now I have to check myself, too (Participant 15).

Spouses also said participating in the program improved their relationship with their wives.

I like these two aspects [taking time to unwind and appreciating her] a lot. It has changed the connection between us in our house and our home in a positive way (Participant 5).

### Improving understanding

Spouses described how much knowledge they gained because of the program. They talked about learning new things.

I think the fact that this gave me the opportunity to learn new things which I didn’t know (Participant 6). and

The program has been an eye opener. There are things I never knew but because of the training I have gained some knowledge (Participant 7).

Spouses mentioned that the new things they had learned from the program improved their understanding of their wives and breast cancer.

A better understanding of what my wife is going through (Participant 10). and

So for me it has improved on my understanding of the breast cancer situation (Participant 7).

### Communicating better

Spouses said the program enabled them to communicate better by improving their communication and helping them listen to their wives.

So, from the beginning of the program to now, I’ll say that it has drastically improved on the way we communicate (Participant 11).

Another spouse said:

The open-ended question. This is a beauty because it takes me out of all the hassle and the struggle, because when I ask why and what, it’s a headache, because you ask one question, you get five questions back. This one is open-ended, and then you just listen. So, it makes it very relaxing, right (Participant 16).

Participant 16 continued to explain the importance of listening because of being in the program:

Not that I don’t listen, but generally given, I’ll say Africans, we don’t listen. We talk past each other. But I’ve seen that the communication in the marriage should completely change when one of you is in this condition. (Participant 16).

## Discussion

Findings from this pilot feasibility study revealed that a 5-session fully scripted intervention delivered in person, over the telephone or zoom, or in a hybrid format was feasible, acceptable, and resulted in improvements in all but one of the standardized measures of spouse caregiver functioning. The program improved spouses’ anxiety, depressed mood, self-efficacy (both wife-focused and self-care subscales) and self-care skills (both wife support and self-care subscales) of spouse caregivers. These improvements show that a structured program for spouse caregivers has the potential to improve their behavioral-emotional adjustment and enhance the quality of their communication with their diagnosed wife. Results also compare favorably to findings from previous studies with primarily White spouses in which the program was delivered in person (Lewis et al. 2008a, 2019). They also compare favorably to results when the intervention was delivered to spouses in small groups (Jones et al. 2013).

Results from spouses’ exit interviews reinforce and expand results from the quantitative measures. Spouses claimed the intervention helped improve their mood and reinforced and enabled them to pay attention to themselves. Prior to the program, spouses reported they did not know they should take care of themselves; rather all attention had been placed on their diagnosed wife. Additionally, they learned how to ask open-ended questions which placed them in a better position to support and communicate better with their wives and improve their relationship. These results are consistent with findings from the pilot feasibility study by Lewis et al. (2008a).

The intervention was feasible despite initial challenges in recruiting eligible participants. Recall that the enrolment rate was 47%. Future studies need to improve this enrollment rate and incorporate additional efficient strategies to recruit spouses, including recruiting from a larger pool of provider agencies. Once recruited, retention was high, 87.5%.

A robust recruitment strategy needs to be developed to enroll a larger and more diverse sample in future studies. Recall that spouses (35%) and their wives (64%) in the current study were from the Akan ethnic group in Ghana, consistent with the 2021 Ghana population census report in which 45.7% of the population were Akans (Ghana 2021 Population and Housing Census 2021). Future studies should enroll more spouse caregivers from the other ethnic groups and spouse participants with lower educational and economic backgrounds.

Future studies need to identify an alternative measure of cancer-related communication between the spouse and wife. Recall that the MIS Scale that was used in the current study failed to show statistically significant changes. These findings may be due to issues of comprehension because spouse participants sought clarification of the meaning of some items on the scale. This nonsignificant result on the MIS is consistent with findings by others (Jones et al. 2013; Lewis et al. 2008a, 2019) but runs counter to what spouses in the current study reported in their exit interviews, namely, that the intervention improved their communication and relationship with their wives.

Spouses in the HHH-Ghana study were more distressed than spouse caregivers enrolled in the HHH-pilot study (Lewis et al. 2008a) (see Table 6). Spouses in the Ghana study had higher scores on both anxiety and depressed mood. We do not know the cause of these elevated scores and are only able to speculate that the financial burden on spouses may be a potential cause. The majority (71%) of spouses mentioned the financial burden on them due to their wives’ breast cancer. Currently, in Ghana, the national health insurance scheme does not cover the full cost of treatment, and spouses must purchase some of the medications. In cases where specific medications are covered by the scheme, the medications are sometimes not available at the health facilities when patients need them. In such cases, spouses must purchase the medication from elsewhere at an increased price.

This study provided preliminary evidence of statistically significant improvements in spouse caregivers’ short-term behavioral and emotional adjustment to their wife’s breast cancer. The program was also acceptable and feasible. Future research is warranted with a longitudinal design that includes a more diverse and larger study sample using mixed-methods with a refined measure of cancer-related couple communication.
